# 原发性纵隔精原细胞瘤伴纵隔肉瘤1例并文献复习

**DOI:** 10.3779/j.issn.1009-3419.2016.09.13

**Published:** 2016-09-20

**Authors:** 进臣 杜, 庆新 李, 栋 晁, 玉莲 班, 群群 李

**Affiliations:** 730050 兰州，兰州军区兰州总医院胸外科 Department of Thoracic Surgery, General Hospital of Lanzhou Army, Lanzhou 730050, China

**Keywords:** 精原细胞瘤, 原发性, 前纵隔, 肉瘤, Primary cell tumor, Primary, Anterior mediastinal, Sarcoma

## Abstract

原发性纵隔精原细胞瘤是极为罕见的性腺外生殖细胞恶性肿瘤，且伴肉瘤成分的纵隔精原细胞瘤更罕见。该病临床表现无特殊性，影像学特征与其他纵隔肿瘤及纵隔型肺癌等难以鉴别，容易误诊。本文详细报道1例原发性纵隔精原细胞瘤伴纵隔肉瘤。通过分析患者诊疗过程，对该病作一综述。

## Abstract (en)

Primary mediastinal seminoma is a kind of germ cell malignancy outside the gonads, and it's rarer with sarcoma component. This disease which has no special clinical manifestations and imaging characteristics is difficult to identify with other mediastinal tumors and mediastinal type lung cancer. This paper reported a case of primary mediastinal seminoma with mediastinal sarcoma. Through the analysis of the diagnosis and treatment process in this patient, we will make a comprehensive review of the disease.

精原细胞瘤多发生于睾丸，是青壮年男性常见的睾丸肿瘤，但仍有约5%-7%发生于性腺器官以外，以纵隔和腹膜后多见。原发性纵隔精原细胞瘤是极为罕见的性腺外生殖细胞恶性肿瘤^[1]^，但伴肉瘤成分的纵隔精原细胞瘤更罕见，目前无相关文献报道。该病临床表现无特殊性，影像学特征与其他纵隔肿瘤及纵隔型肺癌等难以鉴别，容易误诊。兰州军区兰州总医院于2015年9月收治1例原发性纵隔精原细胞瘤伴纵隔肉瘤，为提高对此类疾病的认识及治疗，现结合文献分析报告如下。

## 1 临床资料

### 1.1 一般资料

患者40岁男性，主因“发现右颈部淋巴结肿大2周”入院。查体：右侧锁骨上可触及一肿大淋巴结，大小约3 cm×2 cm，质硬，表面光滑，活动度差，表面无红肿及溃疡，无压痛。前胸壁及颜面部肿胀，前胸壁浅表静脉曲张。胸部X线：前纵隔占位性病变（图 1）。胸部计算机断层扫描（computed tomography, CT）：前纵隔占位性病变，包绕上腔静脉，半包绕升主动脉及主肺动脉上部，考虑为侵袭性胸腺瘤或淋巴瘤；双肺实质内未见明显异常密度影；右侧胸锁乳突肌内侧及锁骨上窝软组织间隙内少量积气（图 1）。实验室检查：甲胎蛋白（alpha fetoprotein, AFP）27.45 ug/L、神经元特异性烯醇化酶（neuron-specific enolase, NSE）82.45 ng/mL、乳酸脱氢酶（lactate dehydrogenase, LDH）989 IU/L、绒毛膜促性腺激素（chorionic gonadotropin, β-HCG）1.2 IU/L、睾酮27.4 nmol/L。

**1:**
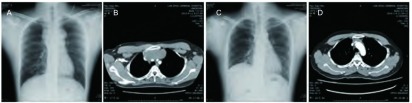
影像学表现。A：中上纵隔占位性病变；B：胸部增强CT检查：前上纵隔占位性病变，包绕上腔静脉，半包绕升主动脉及主肺动脉上部；C：术后复查胸片；D：前中纵隔肿瘤化疗后复查，与前片相比，瘤体有所缩小。 Imaging perfomance. A: Anterior mediastinal space occupying lesion; B: Anterior mediastinal space occupying lesion, superior vena cava, half enclosed in the ascending aorta and upper part of pulmonary artery; C: Postoperative chest X-ray; D: The tumor in the anterior mediastinal were reduced after chemotherapy.

### 1.2 手术与病理

2015年9月8日行右锁骨上淋巴结切除术。术中见右锁骨上区一肿大的淋巴结，大小约3 cm×2 cm，质硬，与周围组织粘连，界限尚清。病理检查：（右锁骨上淋巴结）符合精原细胞瘤。镜下见纤维脂肪中成片的肿瘤细胞生长，瘤细胞圆形或多角形，细胞境界清楚，部分胞浆透亮，核圆形，可见嗜酸大核仁，瘤组织周围见大量的淋巴细胞浸润（图 2）。免疫组织化学检查：CD117(+++)、EMA(++)、CD10(+++)、Ki67≈70%（图 2）；CK18散在(+)、CK19散在(+)、PLAP(+)（图 2）；CKp(-)、TTF-1(-)、MUM1(-)、LCA(-)、HMB45(-)、Vimentin(-)、S-100(-)、ALK(-)、CD45RO(-)、P63(-)、CD5(-)、CK5/ CK6(-)、Bcl-6(-)、CD3(-)、CD20(-)、CD30(-)、CD79a(-)、CD1a(-)。经甘肃省肿瘤医院及北京大学第三医院病理科会诊，均为纵隔精原细胞瘤。术后行全腹核磁提示腹膜后未见明显异常。睾丸彩超检查：双侧睾丸及附睾大小、声像图未见明显异常。证实该病例系原发性纵隔精原细胞瘤，并非性腺或其它部位精原细胞瘤转移所致。术后予以PEB（顺铂20 mg d1-d5，依托泊甙0.2 g d1、d3、d5，博莱霉素60 mg d2、d9、d16）方案联合化疗3次，化疗后复查胸部CT见肿瘤明显缩小（图 1）。于2016年1月12日经胸骨正中切口行纵隔肿瘤切除术。术中见前纵隔一大小约4 cm×3 cm不规则软组织肿块（图 3），质硬，心包、纵隔胸膜及左无名静脉受侵，周围组织水肿明显，质脆，触之易出血。手术沿心底部切开心包，分离肿物与左无名静脉的粘连，左无名静脉侧壁处以5-0无损伤缝线连续缝合，依次切除受侵的胸膜及心包。术后病理：（纵隔）混合性肉瘤，肉瘤成分主要为横纹肌肉瘤和软骨肉瘤，肿瘤性病灶中见多灶性片状分布的凝固性坏死，周围脂性肉芽肿形成，复习前次活检病理诊断为精原细胞瘤，综合两次病理所见，符合伴肉瘤成分的精原细胞瘤，此次化疗后标本经充分取材，仅见残留的肉瘤成分，精原细胞瘤成分再未见到。镜下见：瘤细胞梭形，部分细胞核怪异，间质纤维组织增生，组织细胞及炎细胞聚集，泡沫细胞堆积，见大片坏死区域（图 2）。免疫组化结果：EMA(-)、PLAP(-)、CD117(-)、Ki-67核增殖指数30%、CD10（灶性+）、S100(++)、GFAP(-)、CKp(-)、SMA(-)、Desmin(++)、CD34(-)、Vimentin(+)（图 2）。术后患者恢复顺利，复查胸片未见明显异常（图 1）。术后于2016年2月5日行放射治疗（靶区剂量DT 600 cGy/3次）。术后随访5个月，未发现胸部及睾丸病变。

**2:**
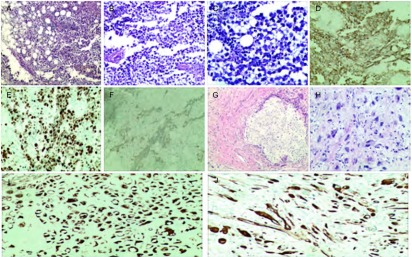
病理图片。A-F：淋巴结病理图片；A-C：HE染色；D-F：免疫组化图片（D: CD117; E: Ki-67; F: PLAP）；G-J：肿瘤病理图片；G-H：HE染色；I-J：免疫组化图片（I: Desmin; J: S-100）（A, G: ×100; B, H: ×200; C: ×400; D, E, F, I, J: ×40）。 Pathology pictures. Pathology pictures of lymph mode (A-F), Haematoxylin eosin staining (A-C) that was positive for CD117 (D), Ki-67 (E), PLAP (F). Tumor pathology pictures (G-J). Haematoxylin eosin staining (G-H) that was positive for Desmin (I), S-100 (J). (A, G: ×100; B, H: ×200; C: × 400; D, E, F, I, J: ×40)

**3:**
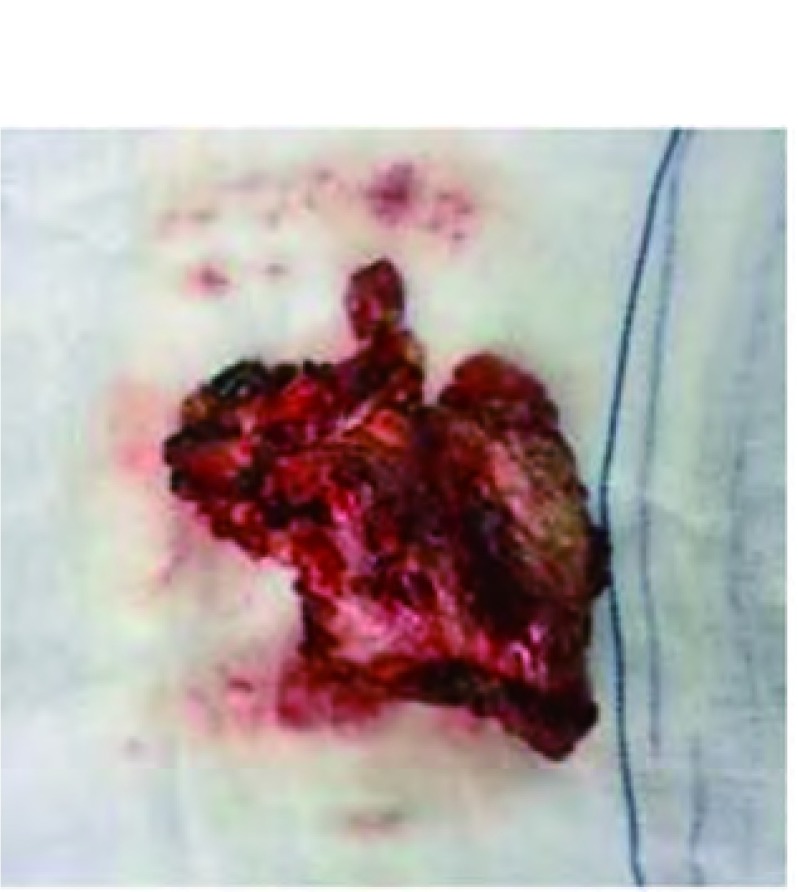
肿瘤大体标本 Gross specimen of tumor

## 2 讨论

原发性纵隔精原细胞瘤由Friedman^[2]^于1951年首次报告，属于性腺外胚胎源性肿瘤，约占纵隔所有肿瘤的0.5%-5%，实属罕见。国内外文献大多是个案报道^[3]^，主要包括精原细胞瘤、胚胎细胞瘤、原发绒癌及卵黄囊瘤^[4]^。该肿瘤发病部位隐匿且生长缓慢，临床及影像学表现均无特异性，术前诊断较困难。由于临床上对此病认识不足，容易误诊或漏诊。本病例首次经锁骨上淋巴结切除后病理诊断为纵隔精原细胞瘤，实验室检查与文献报道一致，术后经多次检查，睾丸未发现异常病灶及异常血流信号，证实本例患者并非性腺生殖细胞肿瘤纵隔转移，系原发于纵隔的精原细胞瘤。虽然精原细胞瘤对化疗非常敏感，化疗后肿瘤明显缩小，但仍有残余肿块。结合化疗后多次胸部CT所见，残余肿块无明显变化。我们认为残存肿瘤对化疗不敏感，单纯放化疗无法达到根治效果，故选择手术切除。对于此类放化疗不敏感的残余肿瘤是否可做为行手术切除的指征，目前尚无相关研究。术后病理检查未见精原细胞瘤成分，仅见残留的肉瘤，结合病理及免疫组化结果，此病例可明确为伴肉瘤成分的精原细胞瘤，即混合型精原细胞瘤。

纵隔精原细胞瘤的发生机理尚不清，一般认为在胚胎发育中，原始生殖细胞在移行过程中迷走或异位到生殖腺外引起^[5]^。发病部位多靠近胸腺，故有学者认为瘤细胞来源于胸腺^[6]^，也有学者认为其系性腺精原细胞瘤转移至纵隔导致^[7]^，但性腺精原细胞瘤很少发生纵隔转移且也可见于女性^[4, 8]^。Sung等^[6]^研究发现纵隔精原细胞瘤患者多存在12p染色体异常。

原发性纵隔精原细胞瘤早期多无症状，20%患者为体检或常规X线胸片发现肿块而就诊^[9, 10]^。症状多出现在病程晚期，临床表现无特异性，主要表现为胸痛、胸闷、气短、咳嗽，重者伴有上腔静脉综合症等全身症状。随着肿瘤增大，发生转移时可出现转移引起的相应症状，如癌性疼痛、胸腔积液及心包积液等。本例患者以右锁骨上淋巴结肿大及上腔静脉阻塞就诊，经右锁骨上淋巴结切除后病理证实为纵隔精原细胞瘤右锁骨上淋巴结转移。影像学主要表现为前纵隔肿块，易与侵袭性胸腺瘤、淋巴瘤或纵隔型肺癌相混淆，亦有文献报道^[8, 11]^发生于中纵隔及后纵隔。X线检查则多表现为纵隔占位性病变，一般位于前纵隔中线偏一侧，向肺内突出，呈半圆形或分叶状（图 1）。胸部CT见肿瘤多发生于前纵隔，沿大血管间隙向四周呈浸润性生长，内部密度不均匀，常有坏死、囊性，但钙化较少见。该肿瘤增强后呈轻、中度强化，周围脂肪间隙消失，肿瘤侵犯纵隔胸膜、心包时可出现胸腔及心包积液^[12]^。覃杰等^[13]^报道精原细胞瘤也可有钙化灶，需于畸胎瘤鉴别。本例患者胸部CT检查符合此特征，但最终诊断仍需依靠病理确诊。实验室检查中纵隔精原细胞瘤可表现为AFP及β-HCG不同程度升高，但无特异性^[14]^。纵隔精原细胞瘤大部分AFP正常，如AFP升高则提示有非精原细胞存在，即混合型精原细胞瘤^[15]^。本例患者检查见AFP明显升高，病理证实为伴肉瘤成分的纵隔精原细胞瘤，符合混合型精原细胞瘤实验室检查。部分病例可见LDH增高，本例与文献报道一致。

Sung等^[6]^研究发现淋巴细胞浸润是纵隔精原细胞瘤最常见的特征。因此，淋巴细胞的存在为精原细胞瘤病理诊断提供了有力支持，但需与淋巴瘤相鉴别。淋巴瘤细胞弥漫分布，较精原细胞瘤小，胞浆少可见淋巴小球^[16]^。镜下见该肿瘤细胞呈多角形，胞浆透明，核圆形，核仁大，有大量淋巴细胞浸润。免疫组化提示PLAP(+)、CAM5.2(+)、HCG(+)、C-kit(+)。有文献^[17]^报道PLAP、CD117、OCT3、OCT4均可表达于精原细胞瘤，精原细胞瘤PLAP(+)表达为80%，CD117和OCT3、OCT4均100%阳性表达。本例免疫组织化学检查CD117(+++)，CD10(+++)及PLAP(+)均呈阳性表达。

原发性纵隔精原细胞瘤为中低度恶性肿瘤，对放疗及化疗敏感，为放化疗可治愈的肿瘤之一^[18]^，预后较好。多选择以顺铂为基础的联合化疗。而精原细胞瘤对放疗也高度敏感，但有学者认为术后放疗有发生重复癌的风险。因此，是否行术后巩固放疗仍待商榷。近来有学者主张单纯使用卡铂化疗可达到和放疗一样的效果^[4]^。通过对本例患者的诊治，我们认为纵隔精原细胞瘤虽对放化疗敏感，但对于放化疗后肿瘤明显缩小或仍有肿块残留时仍应积极手术切除。Hurt等^[19]^认为患者年龄 > 35岁，伴有发热、上腔静脉综合症及锁骨上或颈部淋巴结肿大者多提示预后差。本例患者40岁，入院时已有上腔静脉阻塞及锁骨上淋巴结肿大，术后随访5个月未发现胸部及睾丸病变，远期生存期限及预后有待进一步随访。

综上所述，对于临床上出现的前纵隔肿瘤、呈侵袭性生长、血管间隙周围脂肪组织消失者应考虑纵隔精原细胞瘤。诊断时应重视临床症状、影像及病理检查三者结合。同时应注意鉴别，避免误诊及漏诊。尽早发现并综合治疗，可提高患者生存率，延长生存期。
